# Analysis based on scaling relations of critical behaviour at PM–FM phase transition and universal curve of magnetocaloric effect in selected Ag-doped manganites

**DOI:** 10.1039/c7ra11618g

**Published:** 2018-05-18

**Authors:** S. Tarhouni, R. M'nassri, A. Mleiki, W. Cheikhrouhou-Koubaa, A. Cheikhrouhou, E. K. Hlil

**Affiliations:** LT2S Lab (LR16 CRNS 01), Digital Research Center of Sfax Sfax Technopark, Cité El Ons, B.P. 275 3021 Tunisia; Unité de Recherche Matériaux Avancés et Nanotechnologies (URMAN), Higher Institute of Applied Sciences and Technology of Kasserine, Kairouan University BP 471 Kasserine 1200 Tunisia rafik_mnassri@yahoo.fr; Institut Néel, CNRS et Université Joseph Fourier BP 166 F-38042 Grenoble Cedex 9 France

## Abstract

The critical behaviour of Pr_0.5_Sr_0.5−*x*_Ag_*x*_MnO_3_ (0 ≤ *x* ≤ 0.2) samples around the paramagnetic–ferromagnetic phase transition is studied based on isothermal magnetization measurements. The assessments based on Banerjee's criteria reveal the samples undergoing a second-order magnetic phase transition. Various techniques such as modified Arrott plot, Kouvel–Fisher method, and critical isotherm analysis were used to determine the values of the ferromagnetic transition temperature *T*_C_, as well as the critical exponents of *β*, *γ* and *δ*. The values of critical exponents, derived from the magnetization data using the Kouvel–Fisher method, are found to be (*β* = 0.43 ± 0.002, 0.363 ± 0.068 and 0.328 ± 0.012), (*γ* = 1.296 ± 0.007, 1.33 ± 0.0054 and 1.236 ± 0.012) for *x* = 0.0, 0.1 and 0.2, respectively. This implies that the Pr_0.5_Sr_0.5−*x*_Ag_*x*_MnO_3_ with 0 ≤ *x* ≤ 0.2 systems does not belong to a single universality class and indicates that the presence of magnetic disorder in the system must be taken into account to fully describe the microscopic interaction of these manganites. With these values, magnetic-field dependences of magnetization at temperatures around *T*_C_ can be well described following a single equation of state for our samples. From magnetic entropy change (Δ*S*_M_), it was possible to evaluate the critical exponents of the magnetic phase transitions. Their values are in good agreement with those obtained from the critical exponents using a modified Arrott plot (MAP). We used the scaling hypotheses to scale the magnetic entropy change and heat capacity changes to a universal curve respectively for Pr_0.5_Sr_0.5−*x*_Ag_*x*_MnO_3_ samples.

## Abstract 2

The universal curves of magnetic entropy changes and heat capacity changes for Pr_0.5_Sr_0.5−*x*_Ag*_x_*MnO_3_ (0 ≤ *x* ≤ 0.2) are obtained by using the critical exponents.

## Graphical abstract

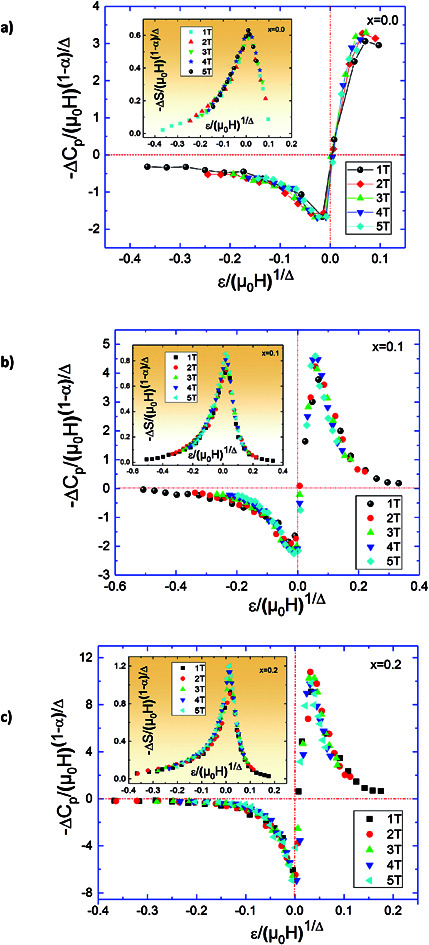

## 1. Introduction

Since the discovery of colossal magnetoresistance (CMR) in perovskite-manganites, extensive efforts have been carried out theoretically and experimentally to investigate the physical properties of these materials.^1–3^ These oxide systems showing multifunctional properties have always had a fascination with science and technology and this has developed into research interests due to the rich fundamental physics and their presence in useful applications including magnetocaloric effect (MCE).^4–6^ This family of materials exhibits a wide variety of interesting phenomena like ferromagnetism, ferroelectricity, transition from metal to insulator behaviour and charge ordering (CO).^7^ The latter properties are related to the competing electron–lattice and electron–electron interactions.^8–10^ Generally, in perovskite-manganites system, studies of divalent cation doping show that manganese ions are formed in two oxidation states: a trivalent state (Mn^3+^) as well as tetravalent state (Mn^4+^). The induced holes in the e_g_ level create a mixed-valence system and it contributed on ferromagnetism and conduction. These behaviours are usually interpreted with the help of double exchange mechanism, where the magnetic coupling between Mn^3+^ and Mn^4+^ ions results from the motion of an electron between the two partially filled d-orbitals with strong on-site Hund's coupling.^11^

The study of phase transitions in manganites is of special importance, since the relationship between the ferromagnetism, CMR and MCE have been a topic of great interest due to the complexity of their magnetic phase diagram. The exploration of critical phenomena in the perovskite manganites has attracted interest since early on in the renaissance of this fascinating class of materials beginning in the 1990s (ref. 12) and still remains one of the actual directions in the condensed state physics. However, half doped manganites Pr_0.5_Sr_0.5_MnO_3_ show interesting properties such as coexistence of contrasting phases, namely, ferromagnetic (FM)–metallic (M) and antiferromagnetic (AF)–insulating (I). In fact, a subtle balance between different phases generally hides behind these complex phenomena, which can be readily shifted or entirely broken by some external stimulus. The substitution with low amount of silver for example destroys the antiferromagnetic phase and drives the system towards a ferromagnetic state at low temperatures.^13^ Meanwhile, it also provides a precious opportunity for us to discover the potential properties and their critical behaviour.

Ferromagnetic Pr-based manganites are intrinsically inhomogeneous, both above and below FM–PM transition temperature (*T*_C_) due to coexistence of FM and antiferromagnetic (AFM) interactions.^14^ Consequently, to get more information about FM–PM transition nature, it is important to study in detail the critical exponents associated with the transition.^15^ This analysis can provide us the order, the universality class, and the effective dimensionality of the phase transition around the Curie temperature *T*_C_.^16^ This practice of assigning such universal classes based on theoretical spin–spin interaction models (like mean field, 3D- Ising or 3D- Heisenberg) has been useful in trying to discern the intricacies of magnetic transitions in real systems.^17^ Several experimental studies of critical phenomena were previously made on ferromagnetic manganites.^18–21^ Therefore, the critical behaviour of manganites near the PM–FM phase transition by using a variety of techniques have yielded a wide range of values for the critical exponent *β*. The values from about 0.3 to 0.5, which embrace mean-field (*β* = 0.5), three-dimensional (3-D) isotopic nearest neighbor Heisenberg (*β* = 0.365) and 3-D Ising (*β* = 0.325) estimates.

In order to establish the relationship between the exponent local *n* and the critical exponents of the materials, field dependence of entropy change (follows a power law Δ*S*^max^_M_ ≈ (*μ*_0_*H*)^*n*^) is checked.^22^ The MCE data of different materials of the same universality class should fall onto the same curve irrespective of the applied magnetic field. Because of the intrinsic relation between the MCE and the universality class, one can obtain the critical exponents based on the MCE data, which may be another method to determine the critical behaviour of phase transition, *i.e.*, the universality class.^23–25^ The present work extends recent studies on Pr-based manganites^26–29^ and presents a detailed investigation of the critical behaviour in Pr_0.5_Sr_0.5−*x*_Ag_*x*_MnO_3_ (0.0 ≤ *x* ≤ 0.2) at its PM–FM transition *via* the detailed measurement of the dc magnetization. The Curie temperature and the critical exponents *β*, *γ* and *δ* were determined. Here the value of field exponent *n* can be obtained from results of the critical behaviour and the MCE analyses. In addition, scaling analysis was used in order to check the validity of these exponents.

## 2. Experimental details

Ceramic samples of Pr_0.5_Sr_0.5−*x*_Ag_*x*_MnO_3_ (0.0 ≤ *x* ≤ 0.2) were synthesized from high purity precursors: Pr_6_O_11_, SrCO_3_, Ag_2_CO_3_ and MnO_2_ (Aldrich 99.9%; USA) in the desired proportions by sol–gel method using nitrate–citrate route.^30^ Stoichiometric amounts of these reagents which were dissolved in diluted nitric acid with continuous stirring and moderate heating resulting in a transparent solution. NH_3_ was added to convert the corresponding precursors into their respective nitrates. Citric acid (C_6_H_8_O_7_) in 1 : 1.5 M ratio with respect to the metal nitrates was added to serve as a complexing agent of the metal ions, and the pH of the solution was controlled by the addition of NH_3_. After continuous stirring for 2 h, ethylene glycol is added as a polymerization agent and the mixture is maintained at 90 °C until the formation of brown gel. The resulting solution was evaporated at 130 °C until a viscous gel-like product was formed. The gel was then dried by slow heating in air up to 300 °C for 2 h. The Pr_0.5_Sr_0.5−*x*_Ag_*x*_MnO_3_ (*x* = 0.0, 0.1 and 0.2) crystallized powders were obtained by calcination at 600 °C for 6 h. The resulting powder was then pressed in a pellet and subsequently heated at 1100 °C for 40 h to be consolidated. Phase purity, homogeneity and cell dimensions were determined by means of X-ray powder diffraction at room temperature (with a diffractometer using Cu Kα radiation). Structural analyses were carried out using the standard Rietveld method.^13,31^ To understand better the critical exponents of the samples accurately, the magnetization as a function of magnetic field for all materials were measured in the range of 0–5*T*. The magnetic measurements were carried out using a “cryogenic” vibrating sample magnetometer (VSM). These isothermals are corrected by a demagnetization factor *D* that has been determined by a standard procedure from low-field dc magnetization measurement at low temperatures (*H* = *H*_app_ − *DM*).

## 3. Results and discussions

X-Ray diffraction study reveals that all samples crystallize in the distorted orthorhombic symmetry with *Pbnm* space group. No impurity peaks were observed in the XRD pattern of the Pr_0.5_Sr_0.5_MnO_3_ sample indicating the single-phase formation of this compound but for the Ag-doped samples a detectable secondary phase Ag-metal appears. According to Rietveld refinement, the cell volume is slightly varying between 228.77 Å^3^ for *x* = 0.0 and 228.9087 Å^3^ for *x* = 0.2 with silver doping and the average crystallite sizes are ∼33.162 nm, 39.0982 nm and ∼59.368 nm respectively for *x* = 0.0, *x* = 0.1 and *x* = 0.2. The morphology and the grains size of the samples reveal that the grains are irregularly spherical-like and the mean sizes are spread between ∼150 nm for *x* = 0.0 and ∼600 nm for *x* = 0.2. This result indicates that the incorporation of Ag into the Sr-site facilities the grain growth during the sintering process. It has been observed that the Ag doping strongly influence the structural. The intrinsic Ag agent enhances the grain size, Mn^4+^/Mn^3+^ ratio, and causes a nonlinear variation in Mn–O–Mn bond angle and electronic bandwidth *W* (see ref. 13 for more details).

**Fig. 1:**
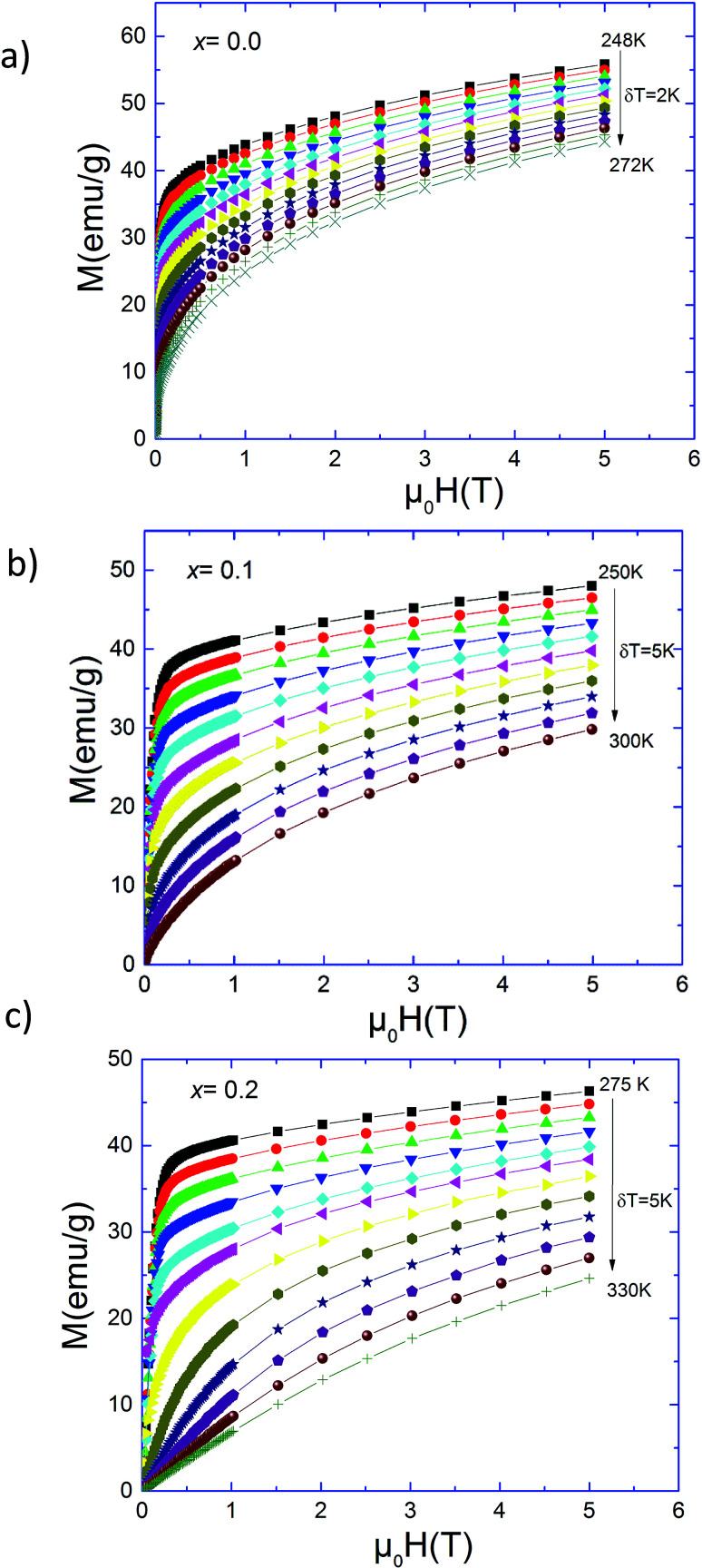
Isothermal magnetization for Pr_0.5_Sr_0.5−*x*_Ag_*x*_MnO_3_ (0 ≤ *x* ≤ 0.2) compounds.

Magnetization measurements *versus* temperature in field cooled (FC) regime of Pr_0.5_Sr_0.5−*x*_Ag_*x*_MnO_3_ (0.0 ≤ *x* ≤ 0.2) samples are reported elsewhere.^13^ Our samples present a transition from a ferromagnetic (FM) to a paramagnetic (PM) state at Curie temperature (*T*_C_). In Fig. 1, we present the isothermal magnetization *M*(*μ*_0_*H*) of Pr_0.5_Sr_0.5−*x*_Ag_*x*_MnO_3_ (0.0 ≤ *x* ≤ 0.2) measured from 0 to 5*T* near their critical regions. These curves exhibit a gradual FM to PM transition. A sharp rise in magnetization at low applied fields has been observed due to magnetic domain rotation in the three compounds.^20^ In the paramagnetic state, the curves *M*(*μ*_0_*H*) begin to be linear only at elevated temperatures, well above the *T*_C_. Below *T*_C_, the magnetization still increases rapidly at low fields, signature of a ferromagnetic behaviour but does not achieve saturation values even at 5*T*.

In order to determine the type of magnetic phase transition in Pr_0.5_Sr_0.5−*x*_Ag_*x*_MnO_3_ (0.0 ≤ *x* ≤ 0.2) samples, we have analyzed Arrott plots which were converted from the isothermal *M*–*μ*_0_*H* data. If long-range interactions were responsible for the ferromagnetic transition, there would be a linear behaviour at high fields around *T*_C_ and the line at *T* = *T*_C_ should just pass through the origin. As all curves present a downward slope, a non-mean field behaviour is present in this case. Following the Banerjee criterion,^32^ the order of magnetic transition can be determined from the slope of straight line. The positive slope corresponds to the second order character of the transition while the negative slope of the curves confirms the first order character transition.^33^

**Fig. 2:**
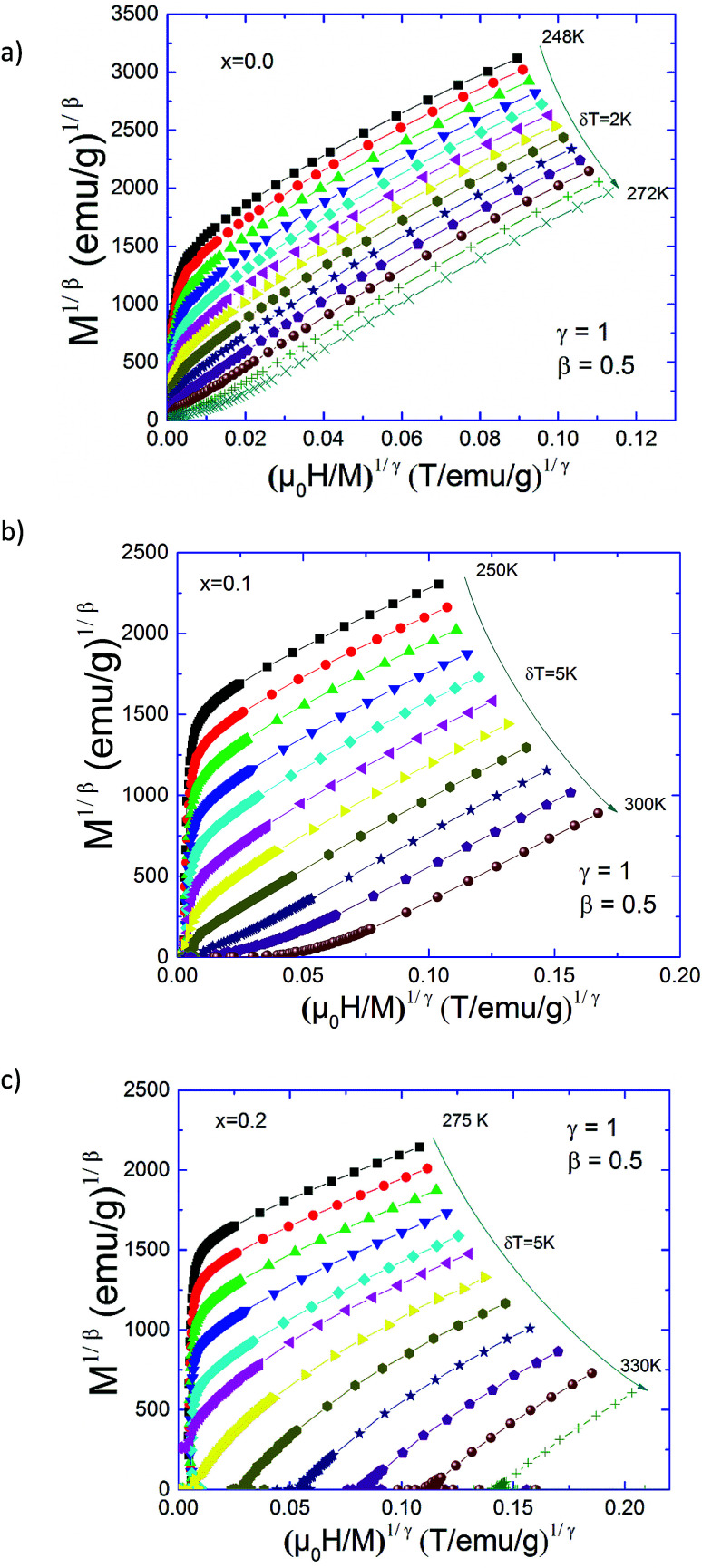
Standard Arrott plots (isotherms *H*/*M vs. M*^2^) for Pr_0.5_Sr_0.5−*x*_Ag_*x*_MnO_3_ (0 ≤ *x* ≤ 0.2) compounds.

Fig. 2 contains the standard Arrott plots constructions where *M*^2^ is represented as a function of *μ*_0_*H*/*M* for Pr_0.5_Sr_0.5−*x*_Ag_*x*_MnO_3_ (0.0 ≤ *x* ≤ 0.2) samples. Positive slopes were observed in the Arrott plots around *T*_C_ for our compounds. Therefore, we can conclude that the para-to-ferromagnetic transition is consistently of second order for all compositions. Basically, the Arrott plots were used to determine the critical exponents if the isotherms *M*^2^*vs. μ*_0_*H*/*M* constitute a set of parallel straight lines around *T*_C_. However, the Arrott plots for our materials show a nonlinear and concave behaviour even in high field range. This indicates that the present phase transition does not satisfy the mean-field theory and that true long-range FM order is absent in all samples. To better, obtain the right values of *β* and *γ* exponents, the mean-field approximation can be generalized to the so-called modified Arrott-plot (MAP) expression, based on the Arrott–Noakes equation of state.^34^ In this context, the second order magnetic transition near the Curie point *T*_C_ is characterized by a set of interrelated critical exponents *β*, *γ* and *δ*.

From magnetization measurements, the mathematical definitions^16,35,36^ of the critical exponents are described below:1
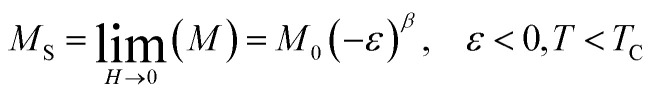
2
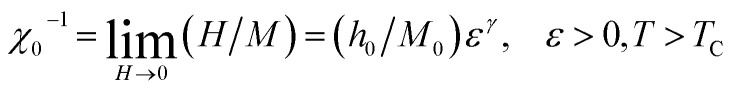

3 *M* = *DH*^1/*δ*^, *ε* = 0, *T* = *T*_C_

where *ε* = (*T* − *T*_C_)/*T*_C_ is the reduced temperature, and *M*_0_, *h*_0_/*M*_0_ and *D* are the critical amplitudes. According, to the prediction of the scaling equation in the asymptotic critical region, the magnetic equation can be given by the following relation:

4 *M*(*H*,*ε*) = *ε*^*β*^*f*_±_(*H*/*ε*^*β*+*γ*^)

where *f*_+_ and *f*_−_ are regular analytic functions for *T* < *T*_C_ and *T* > *T*_C_, respectively. The scaling relation claims that *M*(*H*,*ε*) should yield two universally different branches, one for *T* > *T*_C_ and the other for *T* < *T*_C_. Both these scaling results confirm that the obtained critical exponents and *T*_C_ values are unambiguous, self-consistent and should be intrinsic.

**Fig. 3:**
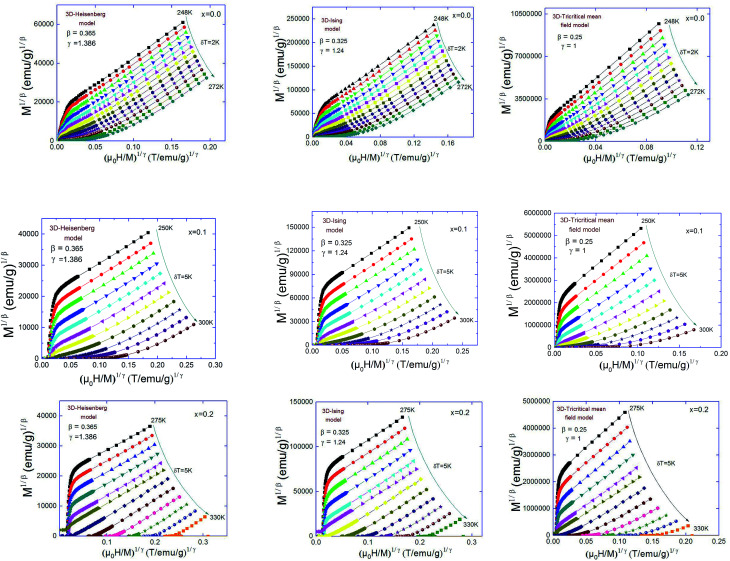
Modified Arrott plots: isotherms of *M*^1/*β*^*vs.* (*H*/*M*)^1/*γ*^ with the 3D-Heisenberg model, the 3D-Ising model and the tri-critical mean-field model for Pr_0.5_Sr_0.5−*x*_Ag_*x*_MnO_3_ compounds.

Generally, the critical exponents and critical temperature can be easily determined from the Arrott–Noakes equation of state:5
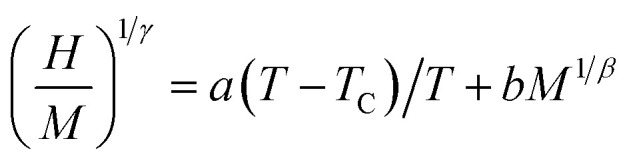
where *a* and *b* are considered to be constants (in the mean-field theory, values of *β* = 0.5 and *γ* = 1 should generate the regular Arrott plots, *M*^2^*vs. H*/*M*). Using different kinds of trial exponents:^37^ 3D-Heisenberg model (*β* = 0.365, *γ* = 1.386), 3D-Ising model (*β* = 0.325, *γ* = 1.241)^38^ and tri-critical mean field (*β* = 0.25, *γ* = 1),^39^ the (*M*)^1/*β*^*vs.* (*H*/*M*)^1/*γ*^ Arrott–Noakes plots are constructed for Pr_0.5_Sr_0.5−*x*_Ag_*x*_MnO_3_ (0.0 ≤ *x* ≤ 0.2) compounds and are shown in Fig. 3.

**Fig. 4:**
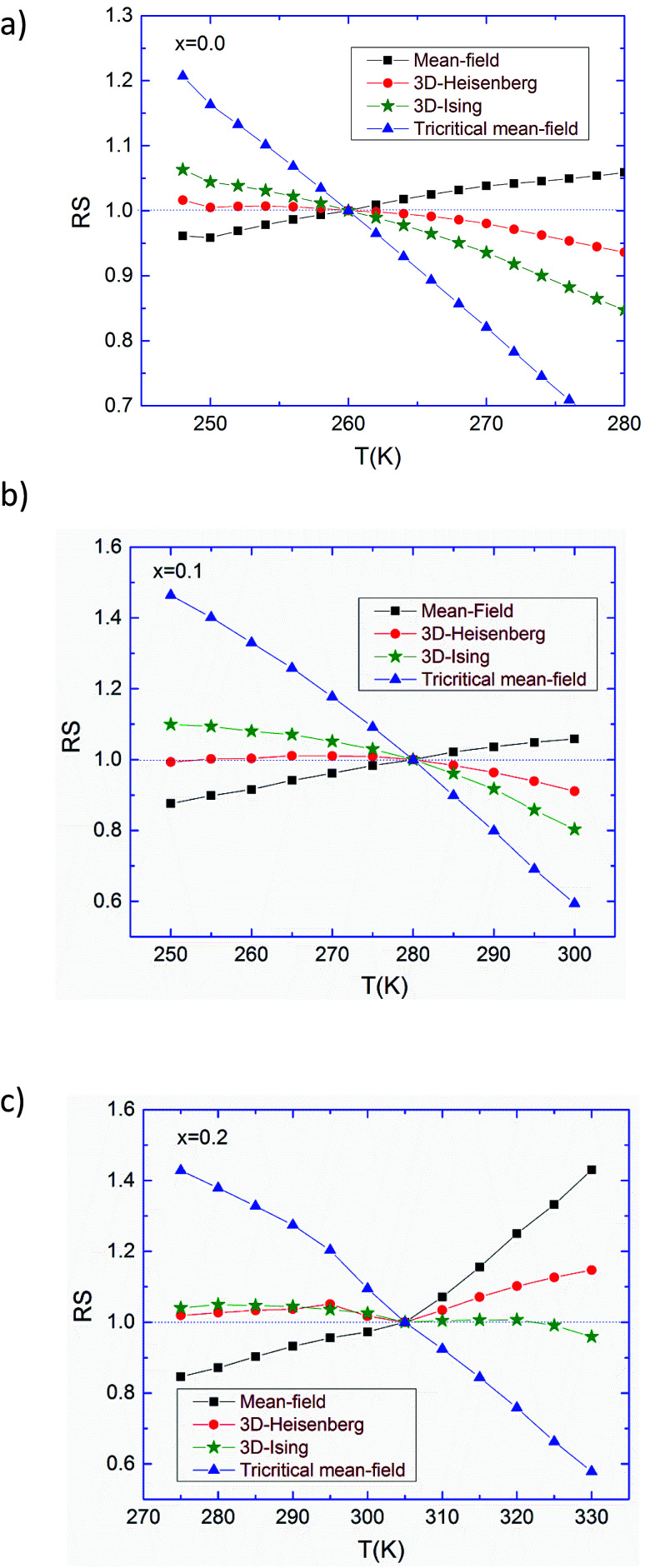
Relative slope (RS) of Pr_0.5_Sr_0.5_MnO_3_ (a), Pr_0.5_Sr_0.4_Ag_0.1_MnO_3_ (b) and Pr_0.5_Sr_0.3_Ag_0.2_MnO_3_ (c) samples as a function of temperature defined as RS = *S*(*T*)/*S*(*T*_C_), using several methods.

These three models yield quasi straight lines and nearly parallel in the high field region. Thus, it is somewhat difficult to determine which one of them is the best for the determination of critical exponents. Thus, we calculated the so called relative slope (RS) defined at the critical point as RS = *S*(*T*)/*S*(*T*_C_). The RS *versus* temperature for the three models is shown in Fig. 4. If the modified Arrott plots show a series of absolute parallel lines, the relative slope of the most satisfactory model should be kept to 1 irrespective of temperatures.^38^ As shown in Fig. 4(a) and (b), the RS of Pr_0.5_Sr_0.5_MnO_3_ and Pr_0.5_Sr_0.4_Ag_0.1_MnO_3_ using mean-field, 3D-Ising and tri-critical mean-field models clearly deviates from 1, but the RS of 3D-Heisenberg model is close to it. As for Pr_0.5_Sr_0.3_Ag_0.2_MnO_3_ it is clearly from Fig. 4(c) the RS for our sample (*x* = 0.2) is very close to 1 when the 3D-Ising model is used. Thus, the critical properties of Pr_0.5_Sr_0.5−*x*_Ag_*x*_MnO_3_ (0.0 ≤ *x* ≤ 0.2) samples can be described with the 3D-Heisenberg for *x* = 0.0 and 0.1 and 3D-Ising models for *x* = 0.2.

**Fig. 5:**
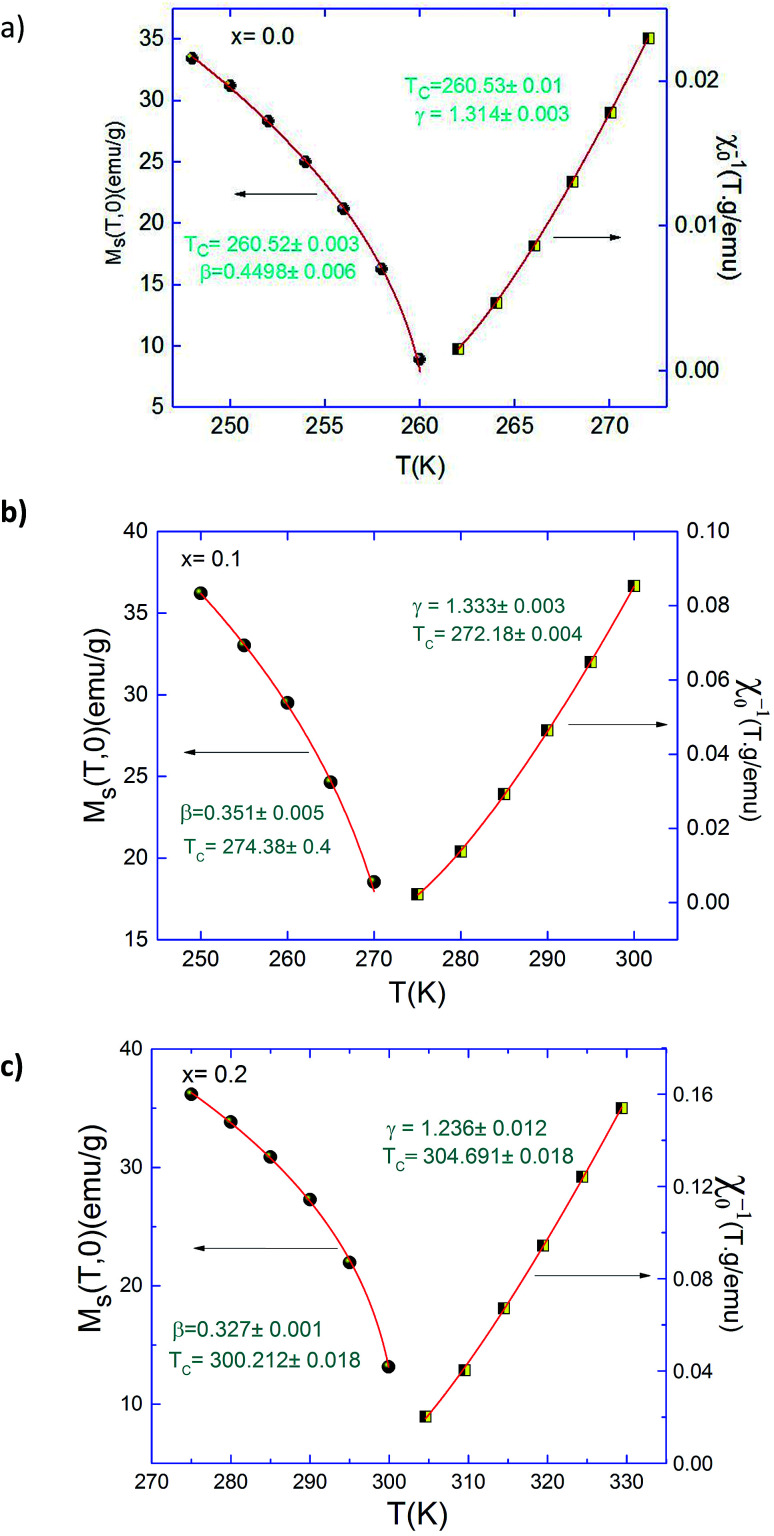
Temperature dependence of the spontaneous magnetization *M*_S_(*T*,0) and the inverse initial susceptibility *χ*_0_^−1^(*T*), along with the fitting curves based on the power laws for Pr_0.5_Sr_0.5−*x*_Ag_*x*_MnO_3_ (*x* = 0.0, 0.1 and 0.2) compounds.

Based on these isotherms, the spontaneous magnetization *M*_S_(*T*,0) and the inverse of susceptibility *χ*_0_^−1^(*T*,0) data can be calculated from the linear extrapolation in the high field straight-line to the co-ordinate axes *M*^1/β^ and (*H*/*M*)^1/γ^, respectively. In Fig. 5 we plotted the temperature dependence of *M*_S_ (*T*,0) and *χ*_0_^−1^(*T*,0) for Pr_0.5_Sr_0.5−*x*_Ag_*x*_MnO_3_ (*x* = 0.1 and 0.2) samples. We get new values of *β*, *γ* and *T*_C_ by fitting these plots with eqn (1) and (2). Thus, new values of the critical exponents were determined and reported in Table 1.

**Table 1:** Comparison of the values of the critical exponents for Pr_0.5_Sr_0.5−*x*_Ag_*x*_MnO_3_ (0 ≤ *x* ≤ 0.2) compounds. With earlier reports, and with the various theoretical models

Materials	Technique	*T*_C_ (K)	*β*	*γ*	*δ*	Ref.
Mean-field model	Theory		0.5	1.0	3.0	18
3D-Heisenberg model	Theory		0.365 ± 0.003	1.386 ± 0.004	4.80 ± 0.04	18
3D-Ising model	Theory		0.325 ± 0.002	1.241 ± 0.002	4.82 ± 0.02	18
Tricritical mean-field model	Theory		0.25	1	5	18
Pr_0.5_Sr_0.5_MnO_3_ (Heisenberg)	MAP	260.52 ± 0.004	0.44 ± 0.006	1.31 ± 0.003	3.98	This work
	KF	260.49 ± 0.03	0.43 ± 0.002	1.296 ± 0.007	4.01	This work
	C.I (exp)	—	—	—	4.383 ± 0.02	This work
Pr_0.5_Sr_0.4_Ag_0.1_MnO_3_ (Heisenberg)	MAP	273.28 ± 0.416	0.351 ± 0.005	1.333 ± 0.0032	4.79	This work
	KF	273.5 ± 0.099	0.363 ± 0.068	1.33 ± 0.0054	4.66	This work
	C.I (exp)	—	—	—	4.037 ± 0.02	This work
Pr_0.5_Sr_0.3_Ag_0.2_MnO_3_ (Ising)	MAP	302.45 ± 0.28	0.327 ± 0.001	1.236 ± 0.012	4.77	This work
	KF	300 ± 0.007	0.328 ± 0.012	1.236 ± 0.012	4.76	This work
	C.I (exp)	—	—	—	3.775 ± 0.03	This work
Pr_0.55_Sr_0.45_MnO_3_		290	0.462	1.033	4.749	46
Pr_0.6_Sr_0.4_MnO_3_		301	0.365 ± 0.004	1.309 ± 0.002	4.648	47
Pr_0.5_Sr_0.5_MnO_3_		261.36	0.443 ± 0.002	1.339 ± 0.006	4.022 ± 0.003	49
Pr_0.52_Sr_0.48_MnO_3_		273	0.462 ± 0.02	1.210 ± 0.03	3.563 ± 0.002	47
Pr_0.8_Na_0.10_K_0.10_MnO_3_	MAP	124.9(1)	0.31(5)	1.29(4)		71
	KF	125.2(6)	0.32(4)			
	C.I (exp)				4.88(2)	
Pr_0.8_Na_0.05_K_0.15_MnO_3_	MAP	134.9(4)	0.32(3)			71
	KF	132.1(2)	0.31(1)			
	C.I (exp)				4.52(1)	
La_0.8_Ca_0.2_MnO_3_		181	0.325	1.180	4.826	48
(Pr,Sm)_0.5_Sr_0.5_MnO_3_		217	0.378	1.2	4.291	20

The best fits of *M*_S_(*T*,0) and *χ*_0_^−1^(*T*,0) give:
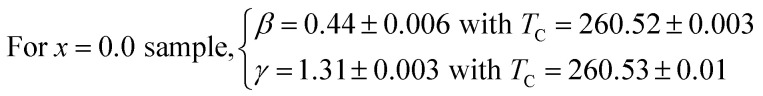

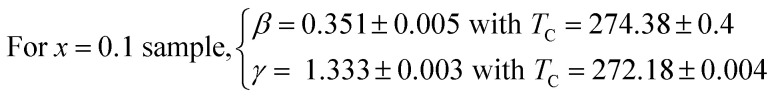

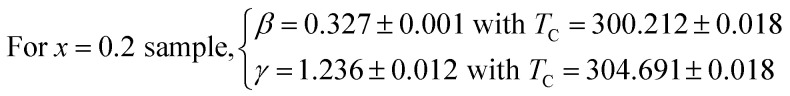


To further support the correctness of the obtained exponents and *T*_C_, Kouvel–Fisher (KF) method^40^ is applied to deduce the critical exponents *β*, and *γ* along with *T*_C_:

6 *M*_S_(*T*)[d*M*_S_(*T*)/d*T*]^−1^ = (*T* − *T*_C_)/*β*

7 *χ*_0_^−1^(*T*)[d*χ*_0_^−1^(*T*)/d*T*]^−1^ = (*T* − *T*_C_)/*γ*

**Fig. 6:**
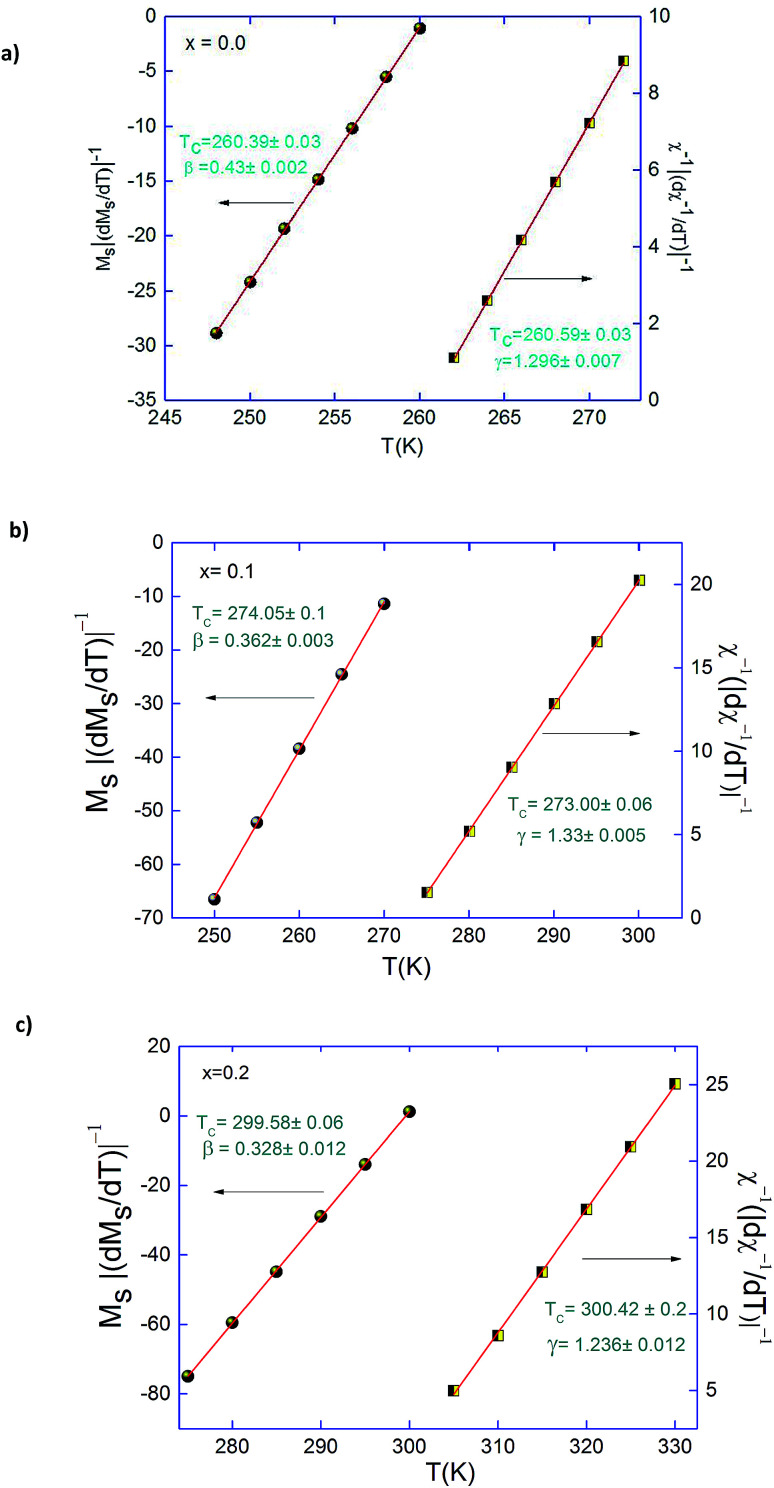
Kouvel–Fisher plots for the spontaneous magnetization and the inverse initial susceptibility for Pr_0.5_Sr_0.5−*x*_Ag_*x*_MnO_3_ (*x* = 0.0, 0.1 and 0.2) compounds.

According to these equations, new plots of *M*_S_ (d*M*_S_/d*T*)^−1^ and *χ*_0_^−1^(d*χ*_0_^−1^/d*T*)^−1^*versus* temperature should yield straight lines with slopes 1/*β* and 1/*γ*, respectively and the intercepts on *T* axes are equal to Curie temperature (*T*_C_). These plots are shown in Fig. 6. The linear fitting to the plots following the KF method gives *β*, *γ* and *T*_C_ which agree well with those that using the MAP of 3D-Heisenberg model for (*x* = 0.0 and 0.1) also 3D-Ising with *x* = 0.2. The critical exponents obtained from KF method are:
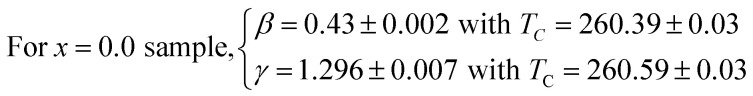

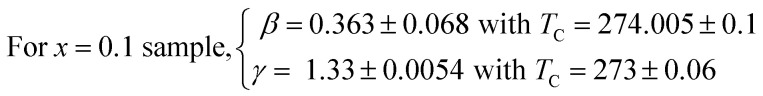

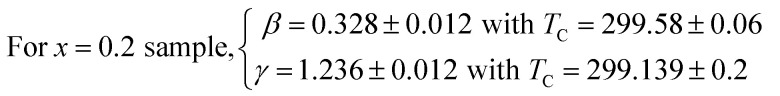


**Fig. 7:**
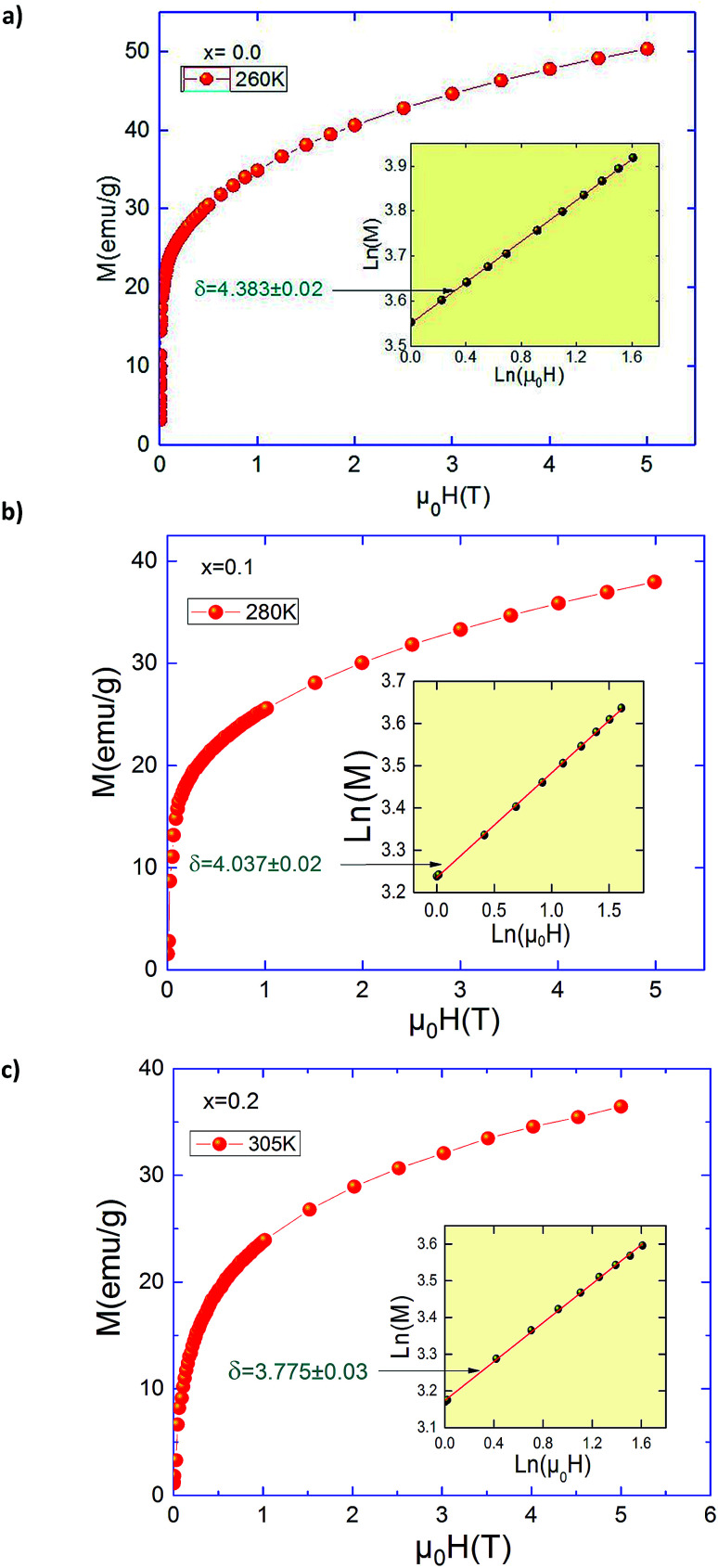
Critical isotherms of *M vs. H* corresponding to *T*_C_ for Pr_0.5_Sr_0.5−*x*_Ag_*x*_MnO_3_ (*x* = 0.0, 0.1 and 0.2) compounds.

Concerning the value of third exponent *δ*, it can be obtained directly by plotting the critical isotherm *M*(*T*_C_,*H*). Fig. 7 shows the critical isotherm *M*(*μ*_0_*H*) curves for *x* = 0.0, *x* = 0.1 and 0.2 measured from 0 to 5*T* at Curie temperatures with a ln(*M*) *vs.* ln(*μ*_0_*H*) scale in its insets. According to eqn (3), ln(*M*) *vs.* ln(*μ*_0_*H*) plot would give a straight line with a slope of 1/*δ*. From the linear fitting, we have got *δ* = 4.383 ± 0.02, 4.037 ± 0.02 and 3.775 ± 0.03 for *x* = 0.0, 0.1 and 0.2, respectively. The exponent *δ* has also been calculated from the Widom scaling relation according to the following equation:^41,42^8
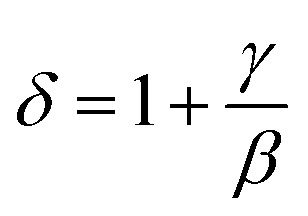


**Fig. 8:**
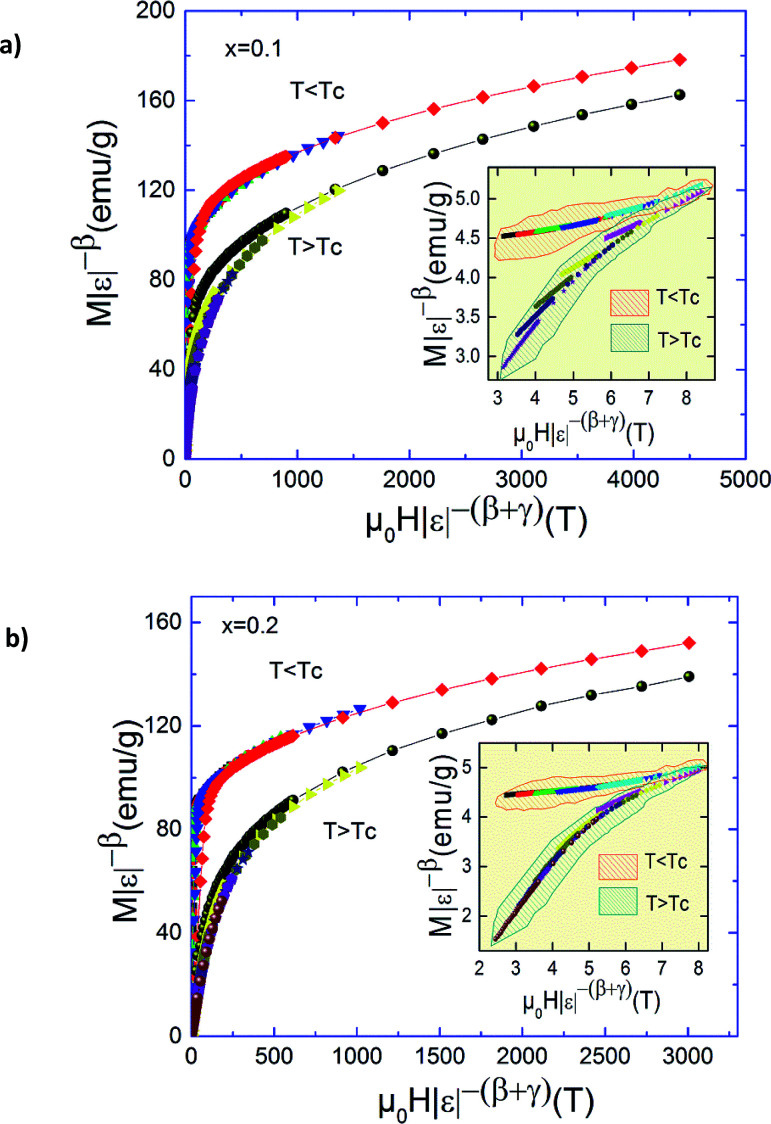
Scaling plots indicating two universal curves below and above *T*_C_ for Pr_0.5_Sr_0.5−*x*_Ag_*x*_MnO_3_ (*x* = 0.1 and 0.2) compounds. Inset shows the same plots on a log–log scale.

Using this scaling relation and the estimated values of *β* and *γ* obtained from the MAP we found: *δ* = 3.98, 4.79 and 4.77 for *x* = 0.0, 0.1 and 0.2, respectively. The values obtained from critical isotherms *M*(*T*_C_,*H*) are slightly larger than determined from the Widom scaling. This difference can be explained by the experiments errors. Therefore, it is important to check if these critical exponents can generate the scaling equation of state for our materials. In the critical region, the magnetization and internal field which should obey the magnetic equation of state is depicted in Fig. 8 for *x* = 0.1 and 0.2, according to eqn (4). The insets show the same plots in log–log scale. It can clearly see that all the points collapse into two different curves, one for *T* < *T*_C_ and the other for temperatures for *T* > *T*_C_. This finding denotes the obtained critical exponents and *T*_C_ are valid. It is found that the scaling is good at higher fields and confirms that the obtained critical exponents and *T*_C_ values are unambiguous, self-consistent and should be intrinsic, whereas the scaling becomes poor, in particular toward low fields which is mainly related to the rearrangement of magnetic domains where magnetic moments are not completely aligned to the field.^43^

Moreover, the critical exponents for the other three compositions with *x* = 0, 0.1 and 0.2 are obtained in the same way, as is summarized in Table 1. For comparison, the predicted values of three classic theoretical models^16,44^ and some of other manganites^18,21,26,45–48^ are also presented in Table 1. A comparison of the current results with the classical models shows that the pristine compound PSMO has the following properties: (i) it does not comply with the 3D-short-range interaction models; (ii) estimated exponents are in between the values for 3D-Heisenberg (*β* = 0.365, *γ* = 1.386) and mean-field model (*β* = 0.5, *γ* = 1) indicating interaction is of extended type; (iii) it is having of itinerant character.^36^ It can be mentioned that the exponents in Table 1 are not consistent with that for dipolar-FM,^49^ so we have attempted to explain the spin interaction in PSMO based on the 3D-Heisenberg model. On the other hand, the critical exponents obtained for Pr_0.5_Sr_0.4_Ag_0.1_MnO_3_ are belong to that expected for the 3D-Heisenberg model (*β* = 0.365, *γ* = 1.386), whereas the values obtained for Pr_0.5_Sr_0.3_Ag_0.2_MnO_3_ are belong to that expected for the 3D-Ising (*β* = 0.325, *γ* = 1.241) model. Generally, 3D-Heisenberg model is useful to describe a magnetic system with isotropic nearest–neighbor exchange interactions between localized spins, and we know that the short-range 3D-Ising model with strong anisotropic properties fully describes the magnetic phase transition. Then, both of which correspond to short range FM interaction.

Physically, *β* describes how the ordered moment grows *T* < *T*_C_ while *γ* describes the divergence of magnetic susceptibility at *T*_C_ and the decrease in *γ* yields a sharp divergence of *χ*(*T*) at Curie temperature. The *β* value decreases with increasing Ag content, reflecting a faster growth of the ordered moment with decreasing temperature in Ag-doped PSMO samples, and is between those expected for the 3D-Heisenberg (*β* = 0.365) and the 3D-Ising (*β* = 0.325) models. This reveals that the short-range FM interaction originates from phase inhomogeneity (due to grain boundaries and isotropic properties) and evolves with addition of disorder depending on Ag concentration. It comes to our attention that the *β* value tends to shift from 0.44 to 0.327 if Ag content increases in PSMO. The fact, that the *β* values are smaller than 0.5 indicates the existence of magnetic inhomogeneity.^50–52^ This microscopic inhomogeneity affects the FM interaction between Mn^3+^ ions to Mn^4+^ ions, and the FM clusters grow in size and number with increasing Ag content, which leads to enhanced PM to FM phase transition in mixed-valence manganites. The Mn^3+^/Mn^4+^ ratio plays a particularly important role in governing the properties of manganites. Indeed, the *β* value tends to shift towards the values of the 3-D Ising model (*β* = 0.325) if the amount of Ag will equal 0.2, indicating the existence of short-range FM order associated with the magnetic inhomogeneity and random distribution of Pr^3+^, Sr^2+^ and Ag^+^ cations with different sizes. Hence, the FM interaction exhibits strong anisotropy, following so the 3D-Ising model for Pr_0.5_Sr_0.3_Ag_0.2_MnO_3_ samples. These results can be explained by the fact that Ag^1+^ ions introduced in the perovskite phase substitutes Sr^2+^ ions resulting some of Mn^3+^ ions to become Mn^4+^ ions proportional the substituted Ag ion concentration. The co-existence of manganese in two valance states plays a critical role on the magnetic properties of these compounds. Mn^3+^/Mn^4+^ ratio changes rapidly by Ag^1+^ ions since one Ag^1+^ oxidizes two Mn^3+^ ions to two Mn^4+^ ions. It is well known that, Mn^3+^ and Mn^4+^ ions differ in their ionic radii. Sr^2+^ and Ag^1+^ ions also have different ionic radii. Because of these ionic radii differences, the lattice parameters of crystal structure of the resulting compound changes, in turn, effecting magnetic properties of the material. The reason for this can be understood considering Zener's double exchange mechanism,^53^ according to which the Mn^3+^/Mn^4+^ ratio and Mn–O–Mn bond length and angle affect the spin ordering of Mn^3+^ and Mn^4+^ ions, resulting a dramatic change in material's magnetic properties.

The obtained value of *γ* increases for *x* = 0.1 and then decreases for *x* = 0.2. This behaviour can be explained by the Ag-doping which has a strongly influence the structural and thermomagnetic properties of the system. The intrinsic Ag agent enhances the grain size, Mn^4+^/Mn^3+^ ratio, and causes a nonlinear variation in Mn–O–Mn bond angle and electronic bandwidth *W*. Therefore, the presence of a maximum in 〈Mn–O–Mn〉 and *W* for *x* = 0.1 has remarkable consequence on the *γ* exponent which exhibits a non-monotonic variation with Ag-doping.

In order to demonstrate the influence of critical exponent on a magnetocaloric effect for a material displaying a second order phase transition, the field dependence of entropy change is analyzed. The field dependence of the peak magnetic entropy change shows a power law

9 Δ*S*_M_ ∞ *H*^*n*^

where the exponent *n* depends on the magnetic state of the compound. It can be locally calculated as follows:10
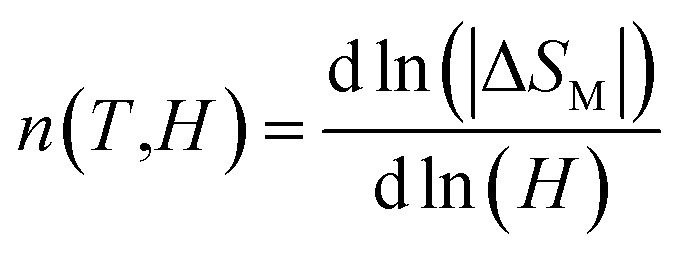


In the particular case of *T* = *T*_C_ or at the temperature of the peak entropy change, the exponent *n* becomes field independent:^54^11
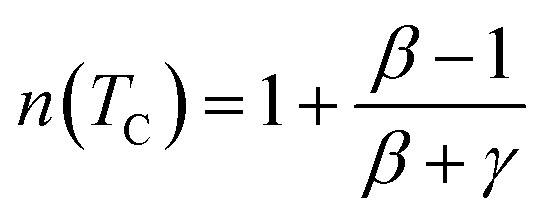
where *β*, *γ* and *δ* are the critical exponents.^38^ With, *βδ* = *β* + *γ* the relation (11) can be written:12
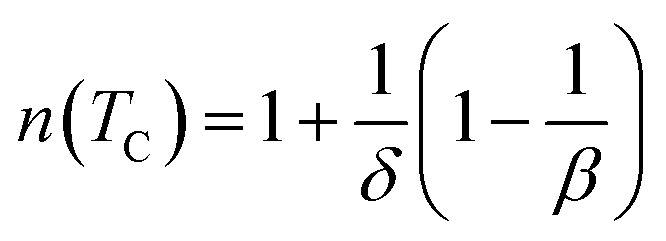


**Fig. 9:**
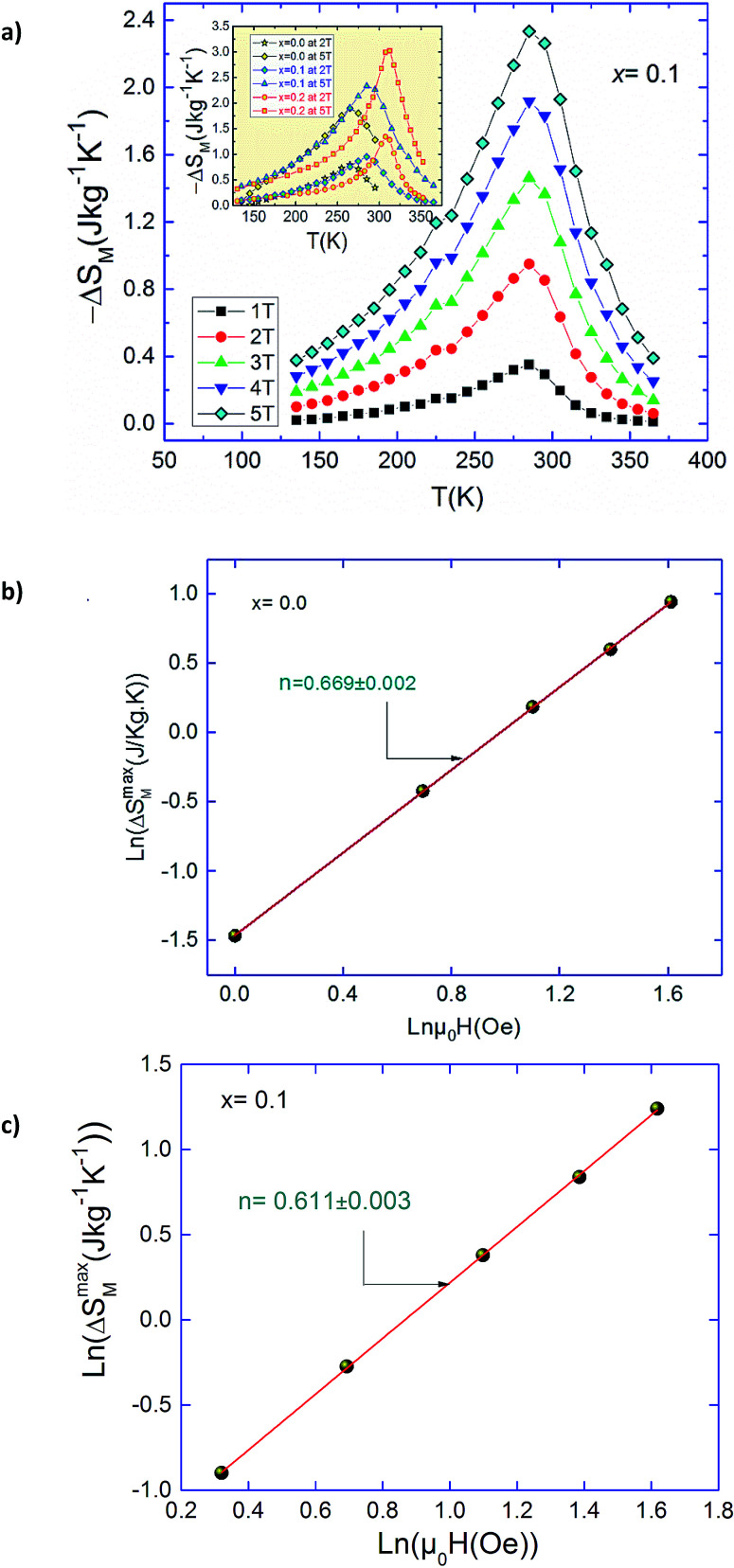
(a) The temperature dependence of the magnetic entropy change for Pr_0.5_Sr_0.5−*x*_Ag_*x*_MnO_3_ (b and c) field dependences of |Δ*S*^max^_M_| at *T*_C_ fitted to a power law Δ*S*^max^_M_ ≈ *a*(*μ*_0_*H*)^*n*^ for Pr_0.5_Sr_0.5_MnO_3_ and Pr_0.5_Sr_0.4_Ag_0.1_MnO_3_ compounds.

Fig. 9a presents the temperature dependence of magnetic entropy change Δ*S*_M_ calculated using the integrated Maxwell relation for *x* = 0.1. The inset of Fig. 9a depicts Δ*S*_M_(*T*) for the three samples near its Curie temperatures under 2 and 5*T*. Fig. 9b and c shows the plot of ln(Δ*S*^max^_M_) *vs.* ln(*H*) at *T*_C_ in relation with eqn (9). The values of *n* extracted from the linear fit of the above plot are *n* = 0.669, 0.611 and 0.572 for *x* = 0.0, 0.1 and 0.2 respectively. Let us recall that the value of *n* obtained from the KF method are about *n* = 0.66, 0.62 and 0.57 for *x* = 0, 0.1 and 0.2 respectively. The nearly equal values obtained from the two methods suggest unambiguously that the obtained coupled values of order parameters are reliable. The obtained values of the local exponent *n* give further insight into the critical behaviour in our samples and the obtained result confirms the presence of the local inhomogeneities in our investigated materials, causing a distribution of Curie temperatures. The mean field approach on the field dependence of the magnetic entropy change at *T*_C_ yields a prediction of *n* = 2/3. One can see that the obtained *n* values presents a small deviation from 2/3 in the exponents. The little difference is ascribed to short-range order related to magnetic disorder and FM clusters existed in the vicinity of a transition temperature.^55,56^ Thus, there are two possible reasons for turning the magnetic interaction from the long-range to the short-range. One is the large spin fluctuation, and the other is the distinct disorder effect. The latter effects are very sensitive to doping element, sintering temperature and preparation method and their changes affect the critical behavior. Several works show that the reduction of grain size and elaborating method strongly influence the universality class.^51,57^ Consequently, the size reduction in system causes a transition phase from a long-range to a short-range order. This observation can be explained by creating some defects at the grain boundaries when the grain size is reduced and the larger surface effects observed in our nano-sized Ag-doped manganites prepared by sol–gel route. Moreover, according to the Harris criterion,^58^ in a second-order phase transition system, if the critical exponent a is positive, the disorder can affect the universality class of the system (as in our case).^59^

According to the renormalization group theory analyses, the universality class for a homogeneous magnet depends on the range of exchange interaction *J*(*r*). Fisher and co-workers^42^ suggest *J*(*r*) = 1/*r*^*d*+*σ*^ where *d* is the dimension of the system and *σ* is the range of exchange interaction. For a three dimension, isotropic system (*d* = 3), the 3D-Heisenberg model (*β* = 0.365, *γ* = 1.386 and *δ* = 4.797) works only if *σ* ≥ 2, *i.e.*, if *J*(*r*) decreases with “short-range” distance faster than *r*^−5^. Whereas if *σ* ≤ 3/2 it complies with the mean-field model (*β* = 0.5, *γ* = 1.0 and *δ* = 3.0), which indicates *J*(*r*) decreases with “long-range” distance slower than *r*^−4.5^. In the intermediate range 3/2 ≤ *σ* ≤ 2, *J*(*r*) decays as ∼*r*^−(3+*σ*)^, the system belongs to different classes with exponents taking intermediate values depending on *σ* value which is the case taking place in our compound for *x* = 0.2.

In order to understand the variation of critical exponents with reduced temperature *ε*, we can determine the effective exponents *β*_eff_ and *γ*_eff_, as per the following relations:13
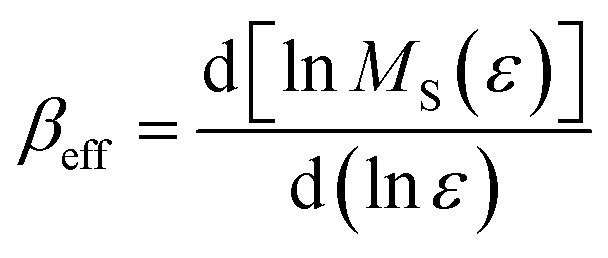
14
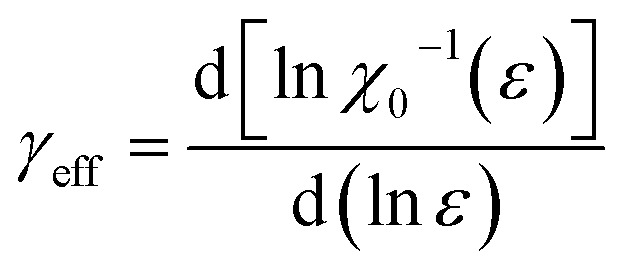


**Fig. 10:**
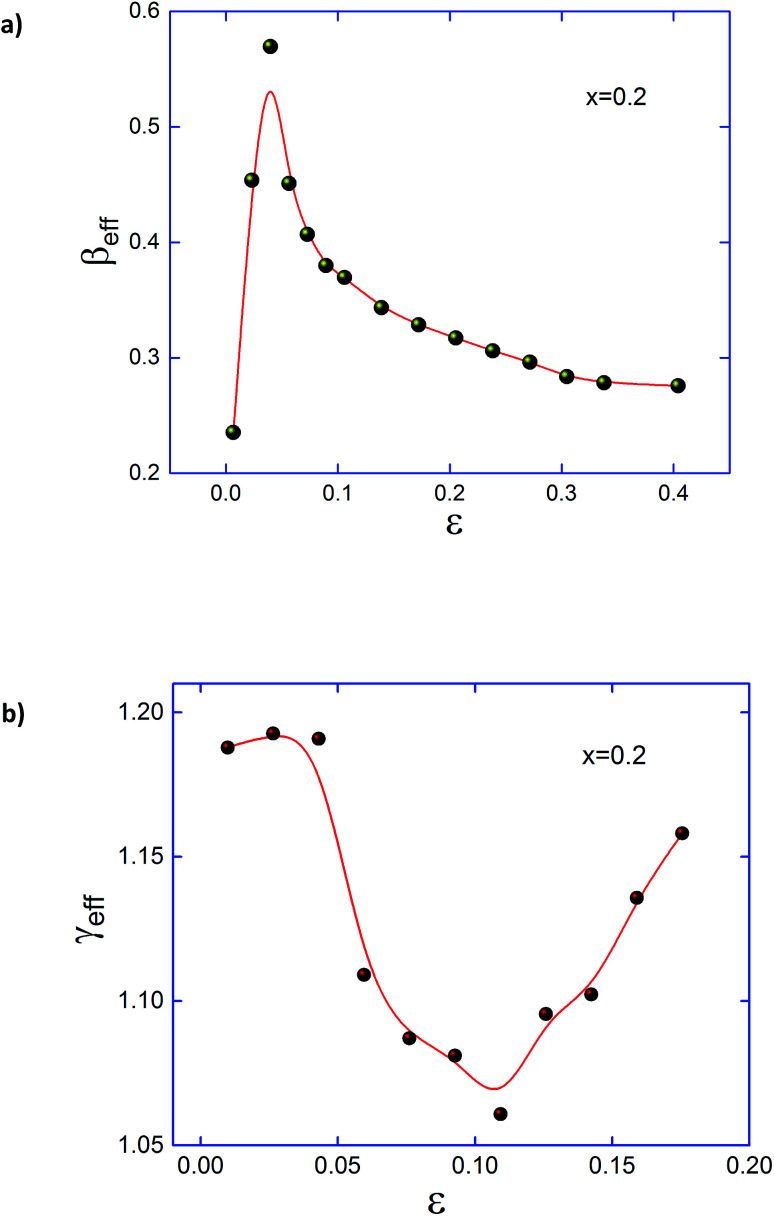
Effective exponents (a) *β*_eff_ below *T*_C_ and (b) *γ*_eff_ above *T*_C_ are plotted as a function of reduced temperature *ε* = (*T* − *T*_C_)/*T*_C_ for Pr_0.5_Sr_0.3_ Ag_0.2_MnO_3_.

Fig. 10a and b shows nonmonotonic changes of the effective exponents *β*_eff_ and *γ*_eff_ as a function of *ε* are plotted for Pr_0.5_Sr_0.3_Ag_0.2_MnO_3_ sample. We conclude from this result that *β*_eff_(*ε*) and *γ*_eff_(*ε*) do not match with any predicted universality class, even in the asymptotic region. Similar phenomenon was found in the pristine compound,^36^ which is considered as characteristic features of a disordered magnet. The magnetic disorder here may result from inhomogeneous magnetic state both below and above *T*_C_ and random distribution of Pr^3+^, Sr^2+^ and Ag^+^ cations with different sizes. However, the critical exponents for a homogeneous ferromagnetic material should be independent of the microscopic details of the system due to the divergence of correlation length in the vicinity of the transition point.^60^ Hence, the critical exponents obtained in Pr_0.5_Sr_0.5−*x*_Ag_*x*_MnO_3_ and the nonmonotonic changes are intrinsic to the system.

Based on the Harris criterion,^58^ if the critical exponent *α*_pure_ > 0, the disorder changes the critical exponents and causes subsequently a crossover to critical behaviour governed by a new random fixed point. While, if the exponent *α*_pure_ < 0, the disorder is irrelevant. Using the Rushbrooke scaling relation expressed as: *α* + 2*β* + *γ* = 2, the exponent *α*_pure_ is found to be positive only for *x* = 0.2 (*α*_pure_ ≈ 0.1), which implies that the disorder is pertinent. This fact explains the obtained values of critical exponents for Pr_0.5_Sr_0.3_Ag_0.2_MnO_3_.

This investigation shows that the Pr_0.5_Sr_0.5−*x*_Ag_*x*_MnO_3_ systems does not belong to a single universality class and the critical behavior is sensitive to the Ag-substitution. Comparing with other Pr-based manganites, the obtained results support evidence of the competition between the short-and long-range ferromagnetic orders. Like results were also observed in Pr_0.15_Ca_0.85_Mn_0.96_Ru_0.04_O_3_ (*β* = 0.478 and *γ* = 1.252),^61^ Pr_0.55_Sr_0.45_MnO_3_ (*β* = 0.462 and *γ* = 1.033),^46^ Pr_0.52_Sr_0.48_MnO_3_ (*β* = 0.462 and *γ* = 1.210),^47^ and so forth (see Table 1). Earlier study^62^ found both narrow-band width Pr-based manganites having the critical exponents close to those expected for the 3D Heisenberg model. Their short-range ferromagnetic interactions are attributed to Ca^2+^ rich regions and established that the Pr_0.7_Ca_0.3_MnO_3_ sample cannot be fitted to the modified Arrott plots due to highly inhomogeneous ground state.^63^ These results show that the critical behavior of Pr_1−*x*_Ca_*x*_MnO_3_ is very sensitive to the amount of calcium which converted the Mn^3+^ ion to the Mn^4+^ ions and modified the Mn–O–Mn networks in this family of materials. It was also suggested that A-site ionic size mismatch may cause a glass-like disorder state, leading to a highly magnetic inhomogeneity.^62^ Recently, Ulyanov and co-workers^64^ have used the electron-spin-resonance spectroscopy for studying praseodymium manganites and found FM and anti-FM interactions associated with both ion types (Pr^3+^ and Mn^3+,4+^) persist in it throughout the temperature range, even above critical temperature. For both wide- and narrow-bandwidth manganites, several investigations of resonant signals (related to the decrease of resonant intensity, the broadening of linewidth, and temperature dependences of the resonant field) in the paramagnetic region show the presence of FM clusters, spin–phonon and/or spin–orbit interactions.^64,65^ These factors may be present in our Pr_0.5_Sr_0.5−*x*_Ag_*x*_MnO_3_, which lead to the magnetic inhomogeneity, and thus influence the critical parameters. It is necessary to emphasize that crystalline characteristics (such as the single crystal and poly-crystalline) also play a significant role affecting the critical behaviour.^66^ Polycrystalline samples with a lower degree of crystalline order normally have a higher magnetic inhomogeneity (due to grain boundaries and isotropic properties), which is absent in single crystals.^62^ Accordingly, magnetic interactions in polycrystalline samples do not completely obey any theoretical model. They are usually mixed by the long-range (corresponding to the MFT) and short-range FM interactions, (corresponding to 3D-Heisenberg and Ising models) and they not belong to a single universality class as in our case.

**Fig. 11:**
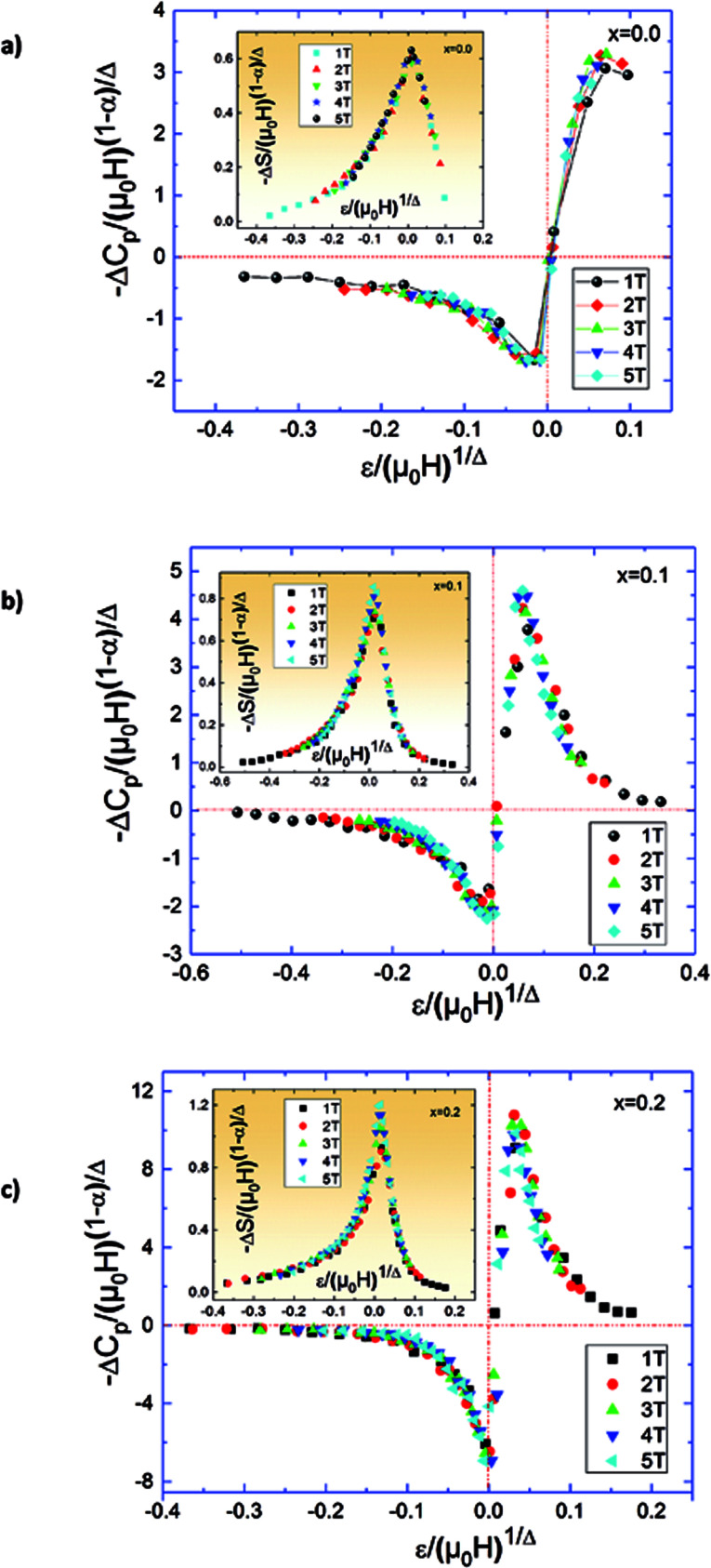
Scaled magnetic entropy changes and heat capacity changes *versus* scaled temperature using critical exponent.

The scaling of Δ*S*(*T*) curves in the vicinity of a second-order phase transition has been theoretically grounded and experimentally confirmed in a variety of magnetic systems.^13,15,23^ In order to continue our investigation in the Pr_0.5_Sr_0.5−*x*_Ag_*x*_MnO_3_ system^13^ and to check the validity of our obtained critical exponents we have using the relation between the critical exponents and the scaled equation of state^67,68^ defined as: 
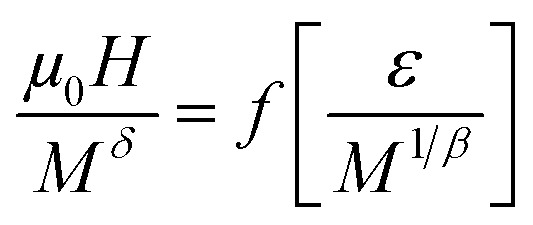
, the obtained Δ*S*(*T*) can be written^69^15
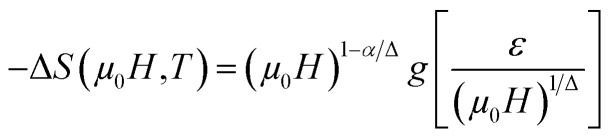
where *α* and Δ are the usual critical exponents. We determine Δ and *α* by using the relations *α* + 2*β* + *γ* = 2 and Δ = *β* + *γ*.^70^Fig. 11 depicts the scaled magnetic entropy change with the scaled temperature. All curves for Pr_0.5_Sr_0.5−*x*_Ag_*x*_MnO_3_ (0.0 ≤ *x* ≤ 0.2) system collapse on a universal curve for several measured fields and temperatures. The good overlap of the experimental data points confirms that the obtained values of *β*, *γ*, and *T*_C_ for this family of manganite are in good agreement with the scaling hypothesis.

The specific heat changes Δ*C*_P_ (not presented here) caused by the applied magnetic field can be calculated from the Δ*S*_M_ entropy change by the following relation:16
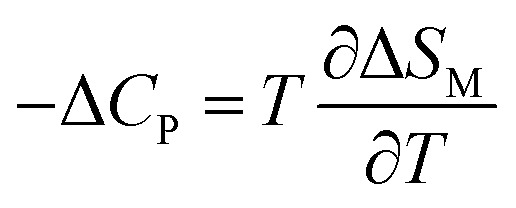


The Δ*C*_P_ for our materials also collapse onto a master curve using the obtained critical exponents. The scaling procedure is a result of the scaling hypothesis for magnetic systems in the critical region. The scaling of Δ*C*_P_(*H*,*T*) as depicted in Fig. 11 shows that 
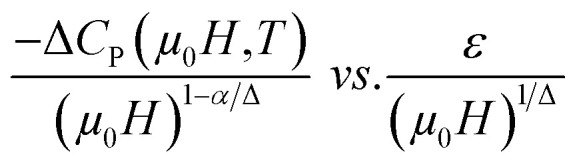
. The excellent overlap of the data points clearly indicates that the obtained values of Curie temperature *T*_C_, *β* and *γ* and for these compounds are in accordance with the scaling hypothesis at several *μ*_0_*H*. The investigation of scaling hypotheses of the thermomagnetic behaviours of Pr_0.5_Sr_0.5−*x*_Ag_*x*_MnO_3_ (0.0 ≤ *x* ≤ 0.2) system gives the possibility of several practical functionality of the universal curve in the characterization of new compounds: as a simple screening process of the performance of samples, as a method for making extrapolations to magnetic fields or temperatures not accessible in the experimental machines, for correcting the influence of non-saturating conditions, for the decrease of the experimental noise, or as a method to eliminate the contribution of minority magnetic phases.

## 4. Conclusion

We studied the critical behaviour of Pr_0.5_Sr_0.5−*x*_Ag_*x*_MnO_3_ (0.0 ≤ *x* ≤ 0.2) nanocrystalline samples around their *T*_C_ values at the PM–FM phase transition. The transition is identified to be second order. The reliable critical exponents *β*, *γ* and *δ* estimated from various techniques such as modified Arrott plot, Kouvel–Fisher method and critical isotherm analysis fit with 3D-Heisenberg for samples (*x* = 0.00 and 0.1) and 3D-Ising for the sample with (*x* = 0.2). The validity of the calculated critical exponents was confirmed using the scaling equation of state. We demonstrate that the critical behaviour of Pr_0.5_Sr_0.5−*x*_Ag_*x*_MnO_3_ is very sensitive to the Ag-doping concentration, which affects both the rate of Mn^3+^/Mn^4+^ conversion and average radius of the A-site cations.

## Conflicts of interest

There are no conflicts to declare.

## Supplementary Material
